# Identification of multi-omics biomarkers and construction of the novel prognostic model for hepatocellular carcinoma

**DOI:** 10.1038/s41598-022-16341-w

**Published:** 2022-07-15

**Authors:** Xiao Liu, Chiying Xiao, Kunyan Yue, Min Chen, Hang Zhou, Xiaokai Yan

**Affiliations:** Department of Oncology, The Second Affiliated Hospital of Zunyi Medical University, Zunyi, 563000 China

**Keywords:** Cancer, Computational biology and bioinformatics, Oncology, Mathematics and computing

## Abstract

Genome changes play a crucial role in carcinogenesis, and many biomarkers can be used as effective prognostic indicators in various tumors. Although previous studies have constructed many predictive models for hepatocellular carcinoma (HCC) based on molecular signatures, the performance is unsatisfactory. Because multi-omics data can more comprehensively reflect the biological phenomenon of disease, we hope to build a more accurate predictive model by multi-omics analysis. We use the TCGA to identify crucial biomarkers and construct prognostic models through difference analysis, univariate Cox, and LASSO/stepwise Cox analysis. The performances of predictive models were evaluated and validated through survival analysis, Harrell’s concordance index (C-index), receiver operating characteristic (ROC) curve, and decision curve analysis (DCA). Multiple mRNAs, lncRNAs, miRNAs, CNV genes, and SNPs were significantly associated with the prognosis of HCC. We constructed five single-omic models, and the mRNA and lncRNA models showed good performance with c-indexes over 0.70. The multi-omics model presented a robust predictive ability with a c-index over 0.77. This study identified many biomarkers that may help study underlying carcinogenesis mechanisms in HCC. In addition, we constructed multiple single-omic models and an integrated multi-omics model that may provide practical and reliable guides for prognosis assessment.

## Introduction

Liver cancer is one of the most prevalent human malignancies globally, seriously threatening people’s lives and health^1^. Hepatocellular carcinoma (HCC) is the predominant liver cancer and accounts for 70–85% of cases^2^. The 5-year survival rate varies greatly in different populations, with an average of about 35%^3–6^. HCC is a highly heterogeneous tumor, and its pathogenesis is quite complicated. Besides, the patients’ outcome is influenced by many factors, such as heredity, environment, and infection. These make the prognosis prediction very challenging^7,8^. Therefore, it is necessary and urgent to develop a robust and practical prognostic evaluation model for HCC.

Previous research has shown that genome changes play an essential role in tumour-related biological processes such as cellular proliferation and differentiation, angiogenesis, stemness, cancer metabolism, immune response, migration, invasion and metastasis^8,9^. Besides, many biomarkers exhibited good prognostic predictive value^10,11^. For example, LMO1 was a critical oncogene that promotes neuroblastoma initiation, progression, and widespread metastatic dissemination^12^. LncRNA SNHG10 was associated with poor overall survival of HCC while influencing the cell proliferation, invasion, migration, cell cycle and epithelial-mesenchymal transition^13^. miR-487a could enhance the proliferation and metastasis of HCC cells by directly binding to sprouty-related EVH1 domain containing 2 (SPRED2) or phosphoinositide-3-Kinase regulatory subunit 1 (PIK3R1) and can be used as a potential prognostic marker^14^. Bezrookove et al. have proved the vital role of PHIP copy-number elevation as a prognostic and progression marker for cutaneous melanoma^15^. SNP in 3′ UTR of RAS-related proteins (RAP1A) was significantly associated with esophageal squamous cell carcinoma risk and metastasis^16^. The continuous discovery of vital biomarkers in various cancers makes up for the inadequacy of traditional predictive models based on clinicopathological characteristics. Therefore, an increasing number of studies are devoted to building predictive models based on genomics.

A comprehensive understanding of human diseases requires the interpretation of molecular intricacy at multiple levels, such as genome, epigenome, and transcriptome. Compared to single-omics analysis, integration of multi-omics data can improve prognostics and predictive accuracy of disease phenotypes by their ability to study the biological phenomenon holistically^17,18^. Because the underlying pathological mechanism of cancer is very complex, the multi‐omics approach is essential for revealing the pathogenic mechanism and evaluating the prognosis^19^. At present, many HCC prediction models based on biomarkers have been reported^20–26^. However, most of them are single-omic models built with RNA-sequence or DNA methylation, and the performance is unsatisfactory, with C-indexes ranging from 0.65 to 0.72. For example, Long et al.’ study^24^ reported a four‐gene‐based prognostic model for HCC with a C-index of 0.65. Even adding the age and pathologic stage information, the C-index is less than 0.70. Such a prediction ability is not excellent. Only Chaudhary et al.^27^ built a multi-omics predictive model with mRNA, miRNA and DNA methylation, which showed a better power than Long et al.’s. However, Chaudhary et al.’s model omitted lncRNA, CNV, and SNP information and is still not very prominent, with a C-index of only 0.70. Therefore, to better evaluate the prognosis and treatment decision-making of HCCs, we tried to construct novel and accurate models through omics features analysis based on mRNA, lncRNA, miRNA, SNP and CNV.

## Results

### Construction and validation of mRNA model

**Figure 1:**
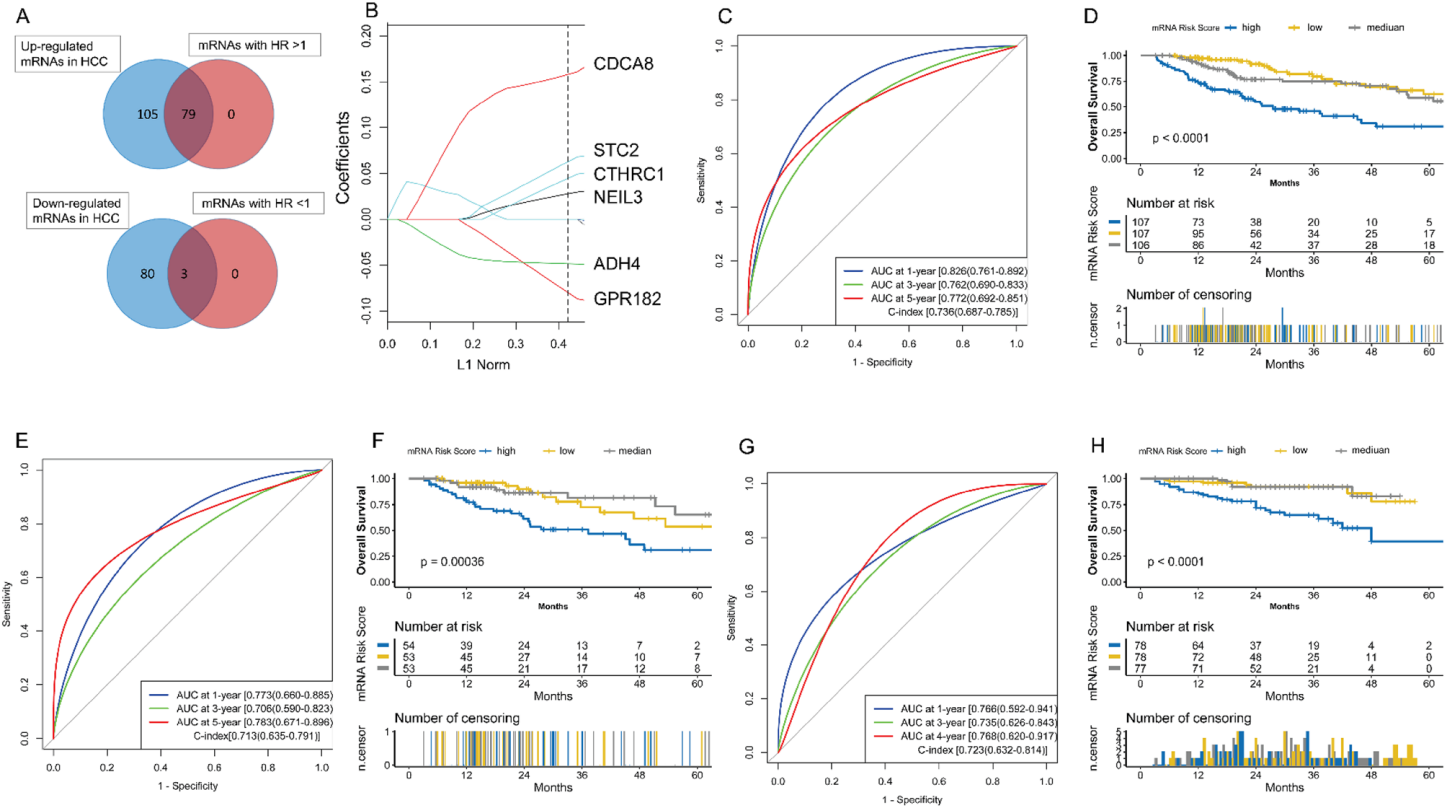
Construction and validation of the mRNA model. **(A)** Selection of mRNAs with HR > 1 and up-regulation, and mRNAs with HR < 1 and down-regulation in HCC. **(B)** LASSO coefficients of the six key mRNAs. The dotted vertical line is drawn at the λ value chosen by the minimum criteria. L1 Norm represents the summation of absolute nonzero coefficients at each λ. Y-axis represents the values of nonzero coefficients at each λ. **(C)** The evaluation of the mRNA model via the ROC curve and C-index in the TCGA training set. **(D)** Kaplan–Meier survival analysis of the different risk groups stratified with the trisection of the mRNA risk score in the TCGA training set. **(E)** The verification of the mRNA model via the ROC curve and C-index in the TCGA test set. **(F)** The verification of the mRNA model with Kaplan–Meier survival analysis in the TCGA test set. **(G)** The external validation of the mRNA model via the ROC curve and C-index in the LIRI-JP dataset. **(H)** The external validation of the mRNA model with Kaplan–Meier survival analysis in the LIRI-JP dataset. *HCC* hepatocellular carcinoma, *TCGA* The Genome Cancer Atlas, *C-index* Harrell’s concordance index, *ROC* receiver operating characteristic, *AUC* area under the curve, *LASSO* least absolute shrinkage and selection operator, *HR* hazard rate ratio.

320 HCC samples with complete mRNA expression profiling and survival information were kept as a training set. 267 DE-mRNAs (including 184 up-regulated and 83 down-regulated mRNAs in HCC) (Fig. S1A,B, Table S1) were selected for univariate Cox regression analysis. Among these DE-mRNAs, 82 mRNAs were significantly associated with OS (Table S2). Then 79 mRNAs with HR > 1 and up-regulated in HCC, and three mRNAs with HR < 1 and down-regulated in HCC were analyzed with LASSO Cox (Fig. 1A). Parameter log (λ) =  − 3.573 (λ = 0.02808) chosen by the tenfold cross-validation method with minimum criteria was regarded as the best value (Fig. S1C). Six key mRNAs with nonzero coefficients (Fig. 1B) were selected to build the mRNA model (Fig. S1F). All were associated with OS (Fig. S1D) and significantly changed in HCC samples (Fig. S1E). The mRNA risk score for each patient was computed: mRNA risk score = ∑βi × exp-mRNA, where exp-mRNA is the expression level of key mRNA and β is the regression coefficient derived from the LASSO COX analysis (Table S9). The mRNA model was evaluated with C-index, ROC curve, and survival analysis (Fig. 1C,D), which showed a relatively good predictive ability (C-index = 0.736). 160 HCC samples were randomly selected as a test set to validate the mRNA model, and good performance was observed (C-index = 0.713) (Fig. 1E,F). The mRNA model was externally verified in the LIRI-JP and GSE1898 datasets, which also showed decent performance (C-index = 0.723) (Fig. 1G,H, Fig. S6A,B).

### Construction and validation of lncRNA model

**Figure 2:**
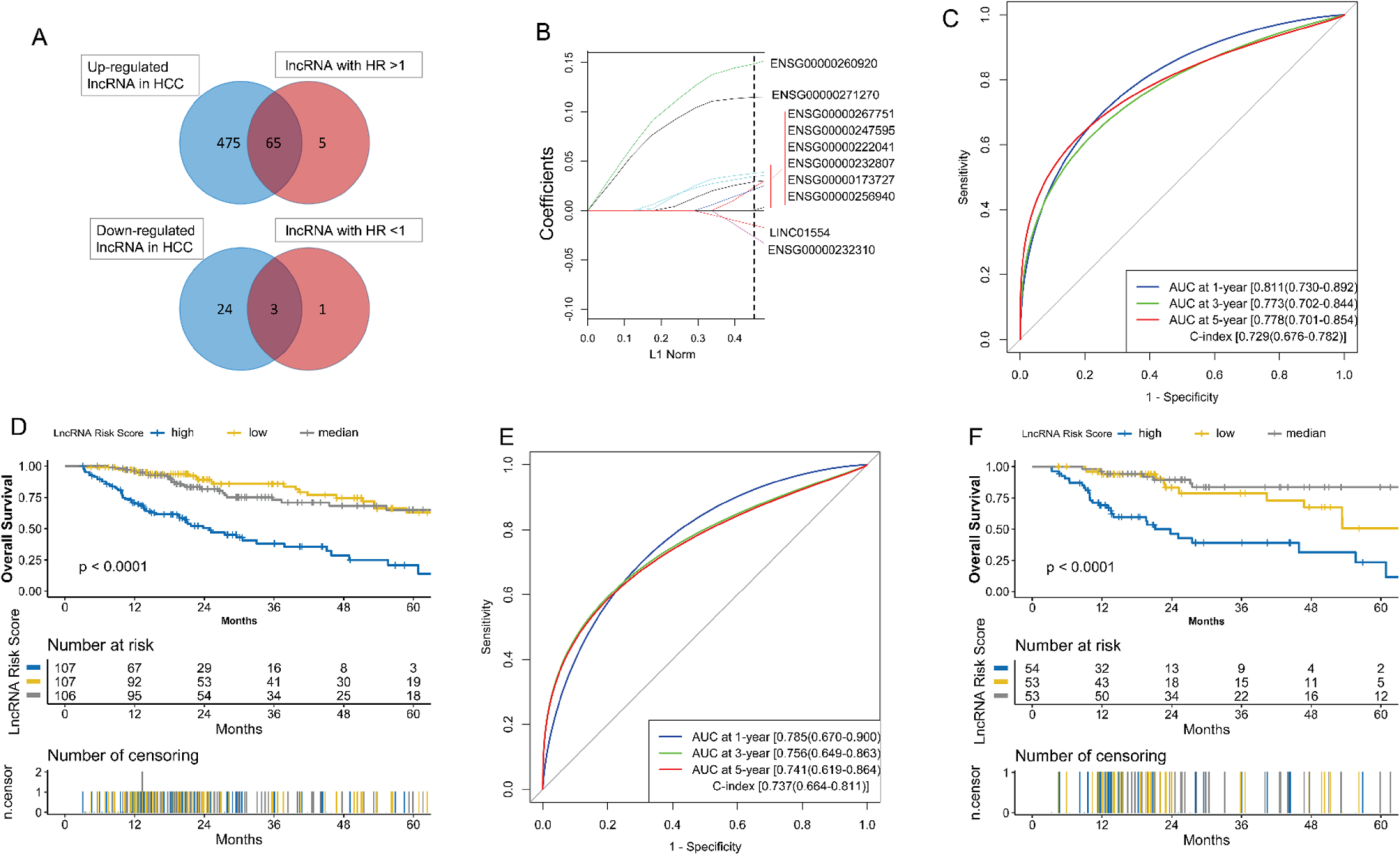
Construction and validation of the lncRNA model. **(A)** Selection of lncRNAs with HR > 1 and up-regulation, and lncRNAs with HR < 1 and down-regulation in HCC. **(B)** LASSO coefficients of the ten key lncRNAs. **(C)** The evaluation of the lncRNA model via the ROC curve and C-index in the TCGA training set. **(D)** Kaplan–Meier survival analysis of the different risk groups stratified with the trisection of the lncRNA risk score in the TCGA training set. **(E)** The verification of the lncRNA model via the ROC curve and C-index in the TCGA test set. **(F)** The validation of the lncRNA model with Kaplan–Meier survival analysis in the TCGA test set. *HCC* hepatocellular carcinoma, *TCGA* The Genome Cancer Atlas, *C-index* Harrell’s concordance index, *ROC* receiver operating characteristic, *AUC* area under the curve, *LASSO* least absolute shrinkage and selection operator, *HR* hazard rate ratio.

320 HCC samples with complete lncRNA expression profiling and survival information were retained as a training set. 540 up-regulated and 27 down-regulated lncRNAs in HCC (Fig. S2A,B, Table S3) were used for Cox regression analysis (Table S4). 68 lncRNAs were selected for LASSO COX analysis (Fig. 2A). Parameter log (λ) =  − 2.621 (λ = 0.07276) chosen by the tenfold cross-validation method with minimum criteria was regarded as the best value (Fig. S2C). Ten key lncRNAs with nonzero coefficients (Fig. 2B) were associated with OS (Fig. S2D) and significantly changed in HCC samples (Fig. S2E), and were used to build the lncRNA model (Fig. S2F). The lncRNA risk score for each patient was computed: lncRNA risk score = ∑βi × exp-lncRNA, where exp-lncRNA is the expression level of key lncRNA, and β is the regression coefficient derived from the LASSO Cox analysis (Table S9). In the training set, the AUC of the lncRNA model at 1, 3, and 5 years OS was 0.811, 0.773, and 0.778, respectively, while the C-index was 0.729 (Fig. 2C). In the test set, the AUC at 1, 3, and 5 years OS was 0.785, 0.756, and 0.741, respectively, while the C-index was 0.737 (Fig. 2E). In addition, the log-rank analysis revealed that scoring using the lncRNA risk score could discriminate the risk groups in the training set and test set (p-value < 0.0001) (Fig. 2D,F).

### Construction and validation of miRNA model

**Figure 3:**
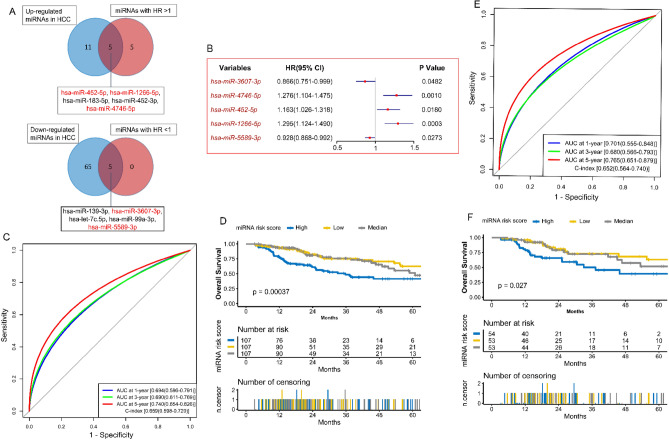
Construction and validation of the miRNA model. **(A)** Selection of miRNAs with HR > 1 and up-regulation, and miRNAs with HR < 1 and down-regulation in HCC. **(B)** Univariate Cox regression analysis of the five key miRNAs. **(C)** The evaluation of the miRNA model via the ROC curve and C-index in the TCGA training set. **(D)** Kaplan–Meier survival analysis of the different risk groups stratified with the trisection of the miRNA risk score in the TCGA training set. **(E)** The verification of the miRNA model via the ROC curve and C-index in the TCGA test set. **(F)** The validation of the miRNA model with Kaplan–Meier survival analysis in the TCGA test set. *HCC* hepatocellular carcinoma, *TCGA* The Genome Cancer Atlas, *C-index* Harrell’s concordance index, *ROC* receiver operating characteristic, *AUC* area under the curve, *HR* hazard rate ratio.

321 HCC samples with complete miRNA and survival information were retained as a training set. Sixteen up-regulated and seventy down-regulated miRNAs in HCC were identified (Fig. S3A, Fig. 2B, Table S5). Ten miRNAs in HCC were used (Fig. 3A, Table S6) for the stepwise Cox analysis, and five key miRNAs were selected to build the miRNA model (Fig. 3B, Fig. S3C,D). The miRNA risk score for each patient was computed: miRNA risk score = ∑βi × exp-miRNA, where exp-miRNA is the expression level of key miRNA, and β is the regression coefficient derived from the stepwise Cox analysis (Table S9). Survival analysis showed that the high-risk group has a poor outcome in the training set (p-value = 0.00037) and test set (p-value = 0.027) (Fig. 3D,F). Besides, in the training and test set, the AUC values of the miRNA model at 1,3, and 5-year points were all more than 0.68, and the C-index values were over 0.65 (Fig. 3C,E).

### Construction and validation of CNV model

**Figure 4:**
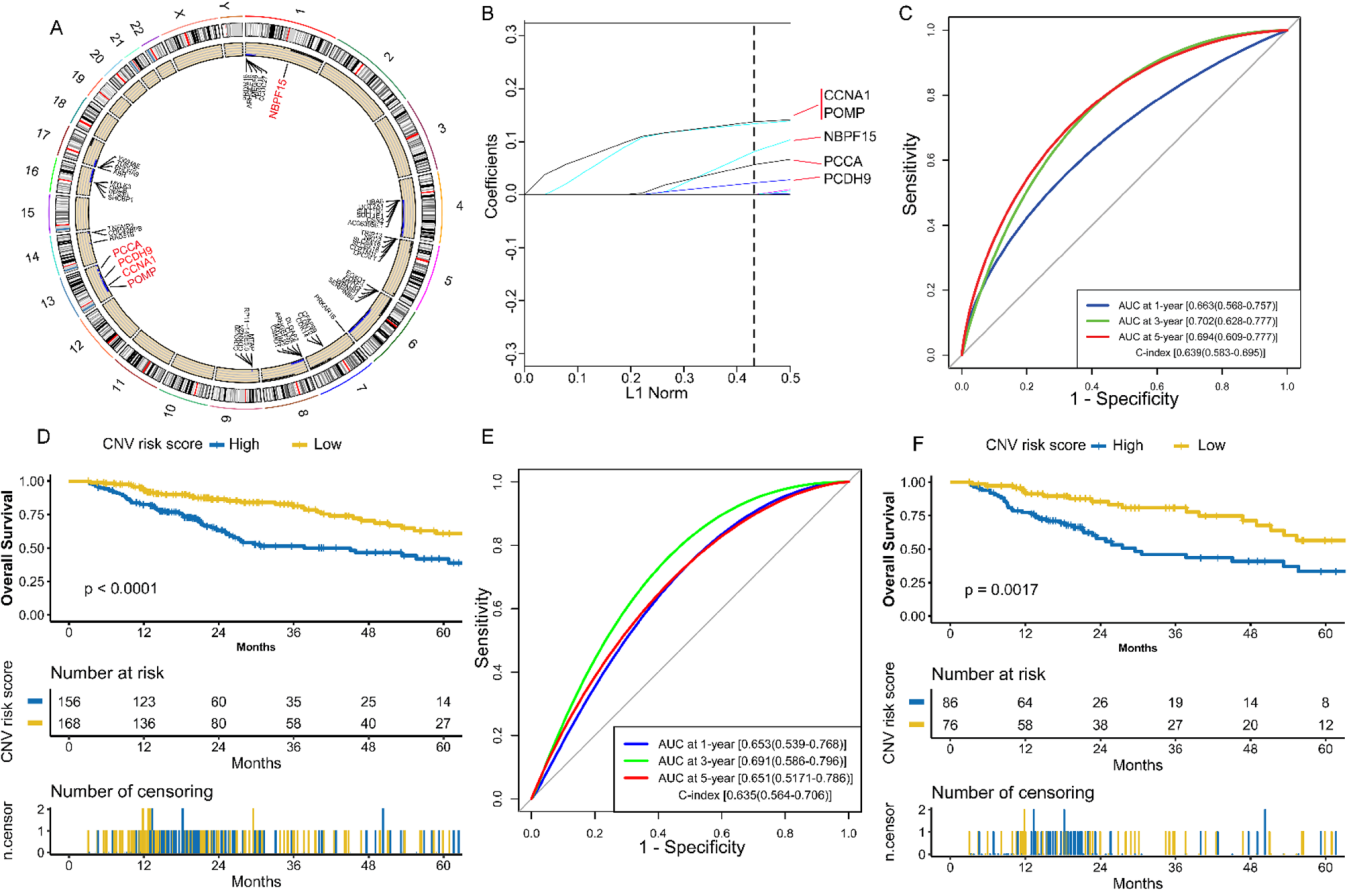
Construction and validation of the CNV model. **(A)** Circos plot shows genes with different copy number alterations between HCC and non-tumor samples. The blue dots represent genes with copy number loss, and the black dots represent genes with copy number gain. **(B)** LASSO coefficients of the five key CNV genes. **(C)** The evaluation of the CNV model via ROC curve and C-index in the TCGA training set. **(D)** Kaplan–Meier survival analysis of the different risk groups stratified with the CNV risk score in the TCGA training set. Patients with no copy number alteration of the five key CNV genes were attributed to the low-risk group and the others to the high-risk group. **(E)** The verification of the CNV model via the ROC curve and C-index in the TCGA test set. **(F)** The validation of the CVN model with Kaplan–Meier survival analysis in the TCGA test set. *HCC* hepatocellular carcinoma, *TCGA* The Genome Cancer Atlas; *C-index*, Harrell’s concordance index, *ROC* receiver operating characteristic, *AUC* area under the curve, *LASSO* Least absolute shrinkage and selection operator, *CNV* copy number variation.

324 HCC samples with complete CNV and survival information were retained as a training set. 5006 genes with different copy number alteration (Fig. 4A, Table S7) were selected to perform univariate Cox regression analysis. 357 CNV genes significantly associated with OS were identified (Table S8). Then we performed LASSO Cox analysis for key CNV genes selection. Parameter log (λ) =  − 2.634269 (λ = 0.07177142) chosen by the tenfold cross-validation method with minimum criteria was regarded as the best value (Fig. S4A). Five key CNV genes with nonzero coefficients (Fig. 4B) were significantly different in HCC samples (Fig. S4B) and associated with OS (Fig. S4C), which were used to build the CNV model (Fig. S4D). The CNV risk score for each patient was computed: CNV risk score = ∑βi × CNV gene status, where β is the regression coefficient derived from the LASSO Cox analysis (Table S9). The CNV model was evaluated with survival analysis in the training set (Fig. 4D) and test set (Fig. 4F), which showed a worse prognosis in the high-risk group (at least one key CNV gene with copy number alteration). Moreover, the AUC values of the CNV model at 1,3 and 5 years OS were all over 0.65, and the C-index values were more than 0.63 (Fig. 4C,E).

### Construction and validation of SNP model

**Figure 5:**
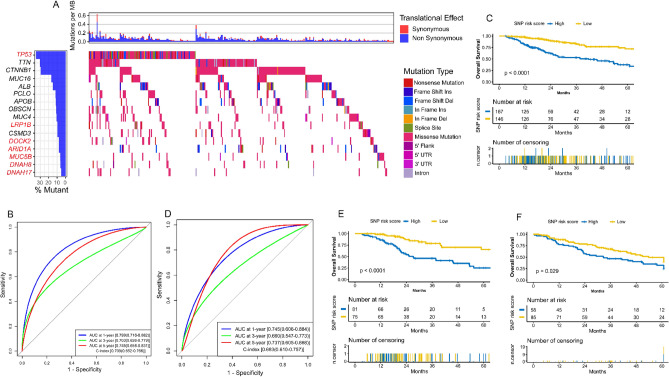
Construction and validation of the SNP model. **(A)** Distributions of various mutation types of the sixteen high-frequency SNPs. The histogram at the top indicates the sum of non-synonymous and synonymous mutations in every case. The histogram on the right stands for the sample number suffering from a gene mutation. The different colors stand for various mutation types in the heatmap, whereas the white represents no mutation. **(B)** The evaluation of the SNP model via the ROC curve and C-index in the TCGA training set. **(C)** Kaplan–Meier survival analysis of the different risk groups stratified with the SNP risk score in the TCGA training set. Patients with no mutation of the seven key SNPs were attributed to the low-risk group, and the others were attributed to the high-risk group. **(D)** The verification of the SNP model via the ROC curve and C-index in the TCGA test set. **(E)** The validation of the SNP model with Kaplan–Meier survival analysis in the TCGA test set. **(F)** The external validation of the SNP model with Kaplan–Meier survival analysis in the LICA-FR dataset. *HCC* hepatocellular carcinoma, *TCGA* The Genome Cancer Atlas; *C-index*, Harrell’s concordance index, *ROC* receiver operating characteristic, *AUC* area under the curve, *SNP* single nucleotide polymorphism.

313 HCC samples with complete SNP and survival information were retained as a training set. Eighty-five high-frequency SNPs (Fig. 5A, Fig. S5A) in HCC were selected to perform univariate Cox analysis, and ten high-frequency SNPs significantly associated with OS were identified (Fig. S5B). Seven key SNPs were selected to build the SNP model through stepwise Cox analysis (Fig. S5C). The SNP risk score for each patient was computed: SNP risk score = ∑βi × SNP status, where β is the regression coefficient derived from the stepwise Cox analysis (Table S9). In the training set, the AUC of the SNP model at 1, 3, and 5 years OS was 0.799, 0.703, and 0.745, respectively, while the C-index was 0.709 (Fig. 5B). In the test set, the AUC at 1, 3, and 5 years OS was 0.745, 0.660, and 0.737, respectively, while the C-index was 0.683 (Fig. 5D). In addition, survival analysis showed that the high risk group (at least one key SNP with non-synonymous mutation) has a poor prognosis in the training set (p-value < 0.0001), test set (p-value < 0.0001), and external validation set (p-value = 0.029) (Fig. 5C,E,F).

### Construction and validation of multi-omics model

**Figure 6:**
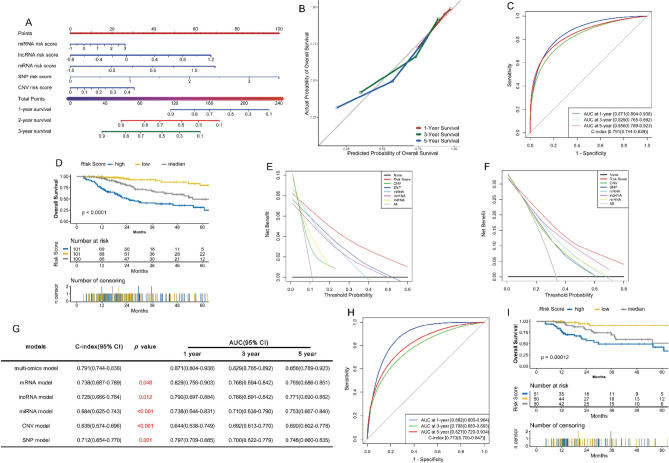
Construction and validation of the multi-omics model. **(A)** Nomogram of the multi-omics model for predicting 1-, 3-, and 5-year OS in the TCGA training set. **(B)** Calibration plot for 1-, 3-, and 5-year OS of the multi-omics model in the TCGA training set. **(C)** The evaluation of the multi-omics model via the ROC curve and C-index in the TCGA training set. **(D)** Kaplan–Meier survival analysis of the different risk groups stratified with the trisection of the total point of the proposed nomogram in the TCGA training set. **(E,F)** Decision curve analysis for the multi-omics model and the five single-omic models at 1- and 3-year points in the TCGA training set. **(G)** Comparison of the predictive power of different models with C-index and ROC analysis in the TCGA training set. **(H)** The verification of the multi-omics model via the ROC curve and C-index in the TCGA test set. **(I)** The validation of the multi-omics model with Kaplan–Meier survival analysis in the TCGA test set. *TCGA* The Genome Cancer Atlas; *C-index*, Harrell’s concordance index, *ROC* receiver operating characteristic, *AUC* area under the curve, *DCA* decision curve analysis, *OS* overall survival, *CNV* copy number variation, *SNP* single nucleotide polymorphism.

302 HCC samples with complete mRNA, lncRNA, miRNA, CNV, SNP, and survival information were retained as a training set. The five single-omic models were integrated through multiple Cox regression analysis to construct a multi-omics model and visualized as a nomogram (Fig. 6A). A fairly good agreement was observed between the expected and observed outcomes for 1, 3, and 5 years OS in the calibration curves (Fig. 6B). Whether in the training set or the test set, the AUC values of the multi-omics model at 1,3, and 5-year points were all over 0.780, while the C-index values were more than 0.770 (Fig. 6C,H), which were significantly greater than those of the five single-omic models (all p values are less than 0.05) (Fig. 6G). DCA analysis showed that the multi-omics model has a better performance in predicting prognosis than the five single-omic models (Fig. 6E,F). In addition, we stratified patients into low, medium and high-risk groups based on the total points of the nomogram (cut-off points were selected at each tertile point). We found that scoring using the nomogram effectively discriminated the risk groups in the training set and test set (p-value < 0.001) (Fig. 6D,I).

## Discussion

With the development of molecular biology techniques, the therapeutic, diagnostic, and predictive value of molecular targets in cancer is gradually becoming evident^28^. Traditional predictive models, such as TNM system^29^, BCLC^30^ and CLIP stage^31^, mainly reflect the clinicopathological characteristics but ignore the genome changes, which are gradually unable to meet the clinical needs in prognosis evaluation. Many HCC prediction models based on biomarkers have been reported^20–26^. However, most of them are single-omic models, with C-indexes ranging from 0.65 to 0.72. Such predictive ability is not satisfactory. Therefore, a more accurate predictive model is needed.

Because multi-omics data can more accurately and comprehensively reflect the widespread biological phenomenon and improve the predictive prognostic accuracy of the disease, we try to construct a robust and efficient prognostic assessment model through multi-omics analysis. This study identified six key mRNAs, ten key lncRNAs, five key miRNAs, five key CNV genes, and seven key SNPs significantly associated with the HCC prognosis. Previous research has demonstrated that most of these critical molecules play essential roles in the occurrence, development, metastasis, and prognosis of HCC. For example, Zhao et al.^32^ have found that NEIL3 could prevent senescence in HCC by repairing oxidative lesions at telomeres during mitosis to promote tumor growth and is significantly associated with poorer survival^33^. CTHRC1 overexpresses in HCC samples, which can promote tumor invasion, proliferation, and motility and predicts poor prognosis^34,35^. STC2 and CDCA8 also have been demonstrated to be significantly associated with the cell proliferation, migration, and growth of HCC, and high expression of them leads to poor overall survival^36–39^. These findings proved that NEIL3, CTHRC1, STC2, and CDCA8 are prognostic risk factors in HCC, which is in line with our analysis (Fig. S1D). Among the ten key lncRNAs, three have been researched in HCC, including LINC01554, CYTOR (ENSG00000222041), and BSG-AS1 (ENSG00000267751). LINC01554 is a novel tumor suppressor that could suppress tumorigenicity in HCC via Akt/mTOR signaling pathway^40^. The down-regulation of LINC01554 significantly predicts worse survival^41^. Ma and Hu et al.^42,43^ have demonstrated that lncRNA CYTOR and BSG-AS1 could promote HCC cell proliferation and growth. Like our analysis (Fig. S2D,E), LINC01554 may function as a tumor suppressor gene, while the CYTOR and BSG-AS1 may act as oncogenes. Three of the critical miRNAs we identified have been reported in HCC. miR-452-5p and miR-1266-5p could mediate the proliferation, migration, and invasion of HCC cell^44,45^, and miR-3607-3p significantly inhibited HCC proliferation and induced apoptosis^46^. In our study, miR-452-5p and miR-1266-5p predict poor survival, while miR-3607-3p acts as benefit factors (Fig. 3B). Among the five key CNVs and seven key SNPs, PCDH9 was reported to inhibit HCC cell proliferation by inducing cell cycle arrest at the G0/G1 phase, and the frequent deletion was observed in Lv et al.’s^47^ and our study (Fig. 4A). Survival analysis in the current study further proves the tumor suppressor function of PCDH9 (Fig. S4C). The frequent mutation of TP53, LRP1B, ARID1A, and DOCK2 in HCC has been confirmed in previous studies^48–51^, which was associated with poor survival, and our research also clarified this point (Fig. 5A, Fig. S5B). All these findings above greatly enhanced the reliability of our analysis results. However, the roles of many vital molecules (e.g., GPR182, ADH4, miR-4746-5p, miR-5589-3p, CNV of CCNA1 and PCCA, ARID1A mutation, etc.) in HCC are still unclear, and further cell and animal experiments to reveal their underlying mechanism is warranted.

Next, we constructed five single-omic predictive models, including mRNA, lncRNA, miRNA, CNV, and SNP. The performance of each single-omic model in prognostic prediction was not bad, with c-index values ranging from 0.63 to 0.73 in the training and test set. Meanwhile, we demonstrated in the separate external validation set that the mRNA and SNP risk scores are significant prognostic factors (Figs. 1G,H, 5F, Fig. S6A,B), which significantly increased the credibility and universality of our analysis results. Of course, compared with other models reported previously^20–26^, our single-omic models have no advantages in prognosis evaluation. Besides, we could not perform the external validation for the lncRNA, miRNA, and CNV models due to the lack of independent external public datasets, which is a shortcoming of our study.

Given that the predictive ability of our single-omic models is not satisfactory, we constructed an integrated multi-omics model based on mRNA, lncRNA, miRNA, CNV, and SNP. The results showed that our multi-omics model has more accurate predictive power than the single-omic models. To the best of our knowledge, our multi-omics model has the most potent predictive ability compared with the previous models based on molecular markers, with a c-index over 0.77 and all AUC values at 1, 3, and 5-years more than 0.78 (Fig. 6C,H). Of course, the lack of external verification is the weakness of this model. To increase the reliability of our research findings, the collection of clinical HCC samples for verification will be the focus of our future work. Besides, our multi-omics model contains more than thirty biomarkers and seems difficult to apply in the clinic. However, more and more patients are willing to use sequencing technology to understand their disease status. Therefore, we believe this model has potential application value in guiding prognostic assessments and treatment decision-making.

In conclusion, the current study identified six key mRNAs, ten key lncRNAs, five key miRNAs, five key CNV genes, and seven key SNPs that are significantly associated with HCC prognosis. These findings may help study underlying carcinogenesis mechanisms in HCC. The predictive models we constructed showed potential prognostic values, which may better guide clinicians in making prognosis assessments and treatment decision-making for HCC patients.

## Materials and methods

### Data acquisition

#### The Genome Cancer Atlas (TCGA)

TCGA (https://portal.gdc.cancer.gov/) is the largest genomic platform for cancer researchers worldwide, covering datasets on 33 different types of cancers and more than 20,000 cancer cases. To perform multi-omics analysis in HCC, we downloaded the mRNA, lncRNA, miRNA, SNP and CNV information from TCGA.

TCGA-LIHC (HCC dataset) was selected in the Project column of the repository interface. The transcriptome profiling, copy number variation, and simple nucleotide variation were selected in the Data Category column. The gene expression quantification, miRNA expression quantification, masked copy number segment, and raw simple somatic mutation were selected as the Data Type. The RNA-seq, miRNA-Seq, WXS, and Genotyping Array were selected in the Experimental Strategy column. The STAR-Counts, BCGSC miRNA Profiling, DNAcopy, and VarScan2 were selected in the Workflow Type column. All the data that matched the above conditions were downloaded. For RNA-Seq data, the raw HTSeq-count data were normalized with the TPM (Transcripts per million) method. Then we obtained the corresponding tissue type, survival time and survival status of HCC from cBioPotal for cancer genomics (https://www.cbioportal.org).

#### The International Cancer Genome Consortium (ICGC)

ICGC (The International Cancer Genome Consortium, https://dcc.icgc.org/releases/current/Projects) is an international project of researcher-generated cancer patient databases. It aims to obtain a comprehensive description of cancer genomic, transcriptomic, and epigenomic changes. In this database, we downloaded two HCC datasets as external validation cohorts to assess the generalizability and accuracy of the mRNA and SNP model, including the LIRI-JP dataset (Liver Cancer-RIKEN, JP project) and the LICA-FR dataset (Liver Cancer-FR project). The mRNA expression data of the LIRI-JP dataset were normalized with the TPM method.

#### GSE1898 dataset

The HCC gene expression dataset (GSE1898) was downloaded from the Gene Expression Omnibus (GEO) (https://www.ncbi.nlm.nih.gov/geo) as an external validation cohort of the mRNA model. The data processing methods were the same as our previous research^11^. The prognostic information of GSE1898 was gained from PRECOG (https://precog.stanford.edu).

### Construction and validation of prognostic models

#### Model based on mRNA expression

All HCC samples of the TCGA dataset were used as a training set. mRNAs expressed in over 95% of samples were retained, and the zero values in the expression matrix were replaced with the minimum non-zero value of the corresponding gene. Then the expression data were log2 transformed. Differentially expressed mRNAs (DE-mRNAs) between HCC and non-tumor samples were identified via ‘limma’ package^52^, and p-value < 0.0001 and |logFC (log fold change)|> 3 were set as the cut-off criteria. Univariate Cox regression analysis was performed to identify mRNAs significantly associated with OS (Overall survival), and a p-value < 0.0005 was considered statistically significant. mRNAs with HR (hazard rate ratio) > 1 and up-regulated in HCC, as well as mRNAs with HR < 1 and down-regulated in HCC, were used for LASSO (least absolute shrinkage and selection operator) COX analysis. Tenfold cross-validation with minimum criteria was applied to identify the optimal parameter λ in LASSO Cox analysis^53,54^ and the key mRNAs. The key mRNAs were used to build a predictive model for HCC. The mRNA risk score for each patient was computed according to the summation of mRNA expression value multiplied by the corresponding coefficient from the LASSO Cox analysis. The performance of the mRNA model in predicting OS was evaluated through survival analysis, Harrell’s concordance index (C-index)^55^, and the receiver operating characteristic (ROC) curve.

50% of HCC samples in TCGA were randomly selected as a test set. Survival analysis, C-index, and ROC analysis were performed to validate the predictive ability of the mRNA model.

The LIRI-JP and GSE1898 were used as independent, external cohorts to assess the generalizability and accuracy of the mRNA model.

#### Model based on lncRNA expression

The methods to construct, evaluate and validate lncRNA model are similar to those in the mRNA model above. To get enough differently expressed lncRNAs (DE-lncRNAs) to establish a stable model, p-value < 0.0001 and |logFC|> 1.5 were set as the cut-off criteria. The p-value < 0.005 was considered statistically significant in the univariate Cox regression analysis. Meanwhile, due to the lack of an external dataset with complete lncRNA expression and corresponding prognostic information, the external verification of the lncRNA model cannot be approached.

#### Model based on miRNA expression

In the TCGA training set, miRNAs expressed in over 80% of samples were retained, and the zero values were processed in the same way mentioned above. ‘limma’ package was performed to identify differentially expressed miRNAs (DE-miRNAs), with a p-value < 0.01 and |logFC|> 1.5. Univariate Cox regression analysis was used to identify miRNAs significantly associated with OS among DE-miRNAs, with a p-value < 0.05. Due to few OS-related miRNAs being obtained, and LASSO Cox is suitable for analyzing high-dimensional data^56^, we used the backward stepwise Cox proportional hazard analysis^57^ to screen critical miRNAs. Then the same methods used in the mRNA model were performed to construct, evaluate, and validate the miRNA model. For the same reason, we cannot complete the external verification of the miRNA model.

#### Model based on CNV

In the TCGA training set, the segment mean value is used to reflect the CNV of DNA fragments. A segment is called a gain or loss if the segment mean value is more or less than zero. According to the GENCODE v34 annotation file (downloaded from https://www.gencodegenes.org) and segment mean value of DNA fragments, we identified genes with copy number variation (CNV genes) in each sample. Chi-square analysis was used to compare the statistical difference of CNV genes between HCC and non-tumor samples. Then we used the univariate Cox regression analysis to identify CNV genes significantly associated with OS. The LASSO Cox analysis was used to screen key CNV genes and construct the CNV model. The CNV risk score for each patient was computed according to the summation of CNV gene status (non-CNV = 0; CNV = 1) multiplied by the corresponding coefficient from the LASSO Cox analysis. The evaluation and validation methods of the CNV model are the same as those in the mRNA model. We could not perform external validation for the CNV model for the same reason.

#### Model based on SNP

In the TCGA training set, the high-frequency SNPs (not including synonymous mutation) in HCC samples were selected to perform the univariate Cox analysis. Due to few OS-related SNPs being obtained, we performed the backward stepwise Cox proportional hazard analysis to identify critical SNPs and build the SNP model. The SNP risk score for each patient was computed according to the summation of SNP status (wild = 0; mutation = 1) multiplied by the corresponding coefficient from stepwise Cox analysis. Then the same methods we used in the mRNA model were performed to evaluate and validate the SNP model. The LICA-FR dataset was used as an independent, external cohort to assess the generalizability and accuracy of the SNP model through survival analysis.

#### Model based on multi-omics

**Figure 7:**
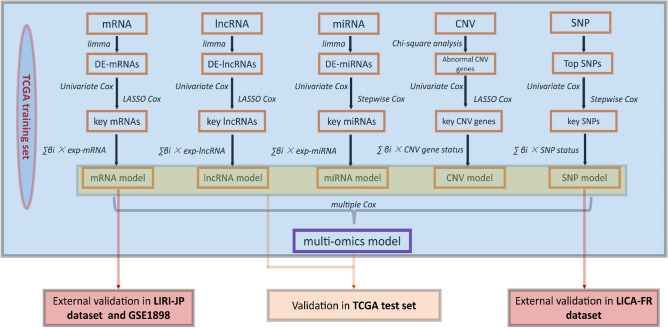
Overall workflow. We used all HCCs in TCGA as a training set and 50% of HCCs as a test set. In the training set, we performed the limma analysis to identify DE-mRNAs, DE-lncRNAs, and DE-miRNAs. Chi-square analysis was used to screen abnormal CNV genes. The high-frequency SNPs (Top SNPs) in HCC were selected for further research. The univariate Cox regression analysis, LASSO Cox analysis, and backward stepwise Cox proportional hazard analysis were used to identify critical markers. We constructed five single-omic models (mRNA, lncRNA, miRNA, CNV, and SNP model) through LASSO Cox analysis or stepwise Cox. The multi-omics model was constructed based on the five single-omic models through multiple Cox regression analysis. These models were evaluated and verified in the training set and test set, respectively. Moreover, we externally validated the mRNA and SNP models in the LIRI-JP, GSE1898, and LICA-FR, respectively. *HCC* hepatocellular carcinoma, *TCGA* The Genome Cancer Atlas, *LASSO* Least absolute shrinkage and selection operator, *OS* overall survival, *DE-mRNAs* Differentially expressed mRNAs, *DE-lncRNAs*, differently expressed lncRNAs, *DE-miRNAs* differentially expressed miRNA, *CNV* copy number variation, *SNP* single nucleotide polymorphism.

We built a multi-omics model based on the mRNA, lncRNA, miRNA, SNV, and SNP risk scores through multiple Cox regression analyses. Nomogram was used for the visualization of the prediction model. We performed the survival analysis, calibration plot, C-index, ROC, and decision curve analysis (DCA) to evaluate and compare the predictive ability of the multi-omics model with the five single-omic models. We performed the same methods in the mRNA model to validate the multi-omics model in the test set. The entire workflow is shown in Fig. 7.

### Statistical analysis

We performed data processing and statistical analysis with R (https://www.r-project.org/, v 3.6.0). Chi-square or Fisher’s exact test was used to assess differences in categorical variables. Student t-test or non-parametric Mann–Whitney test was used to detect differences in continuous variables. Volcano, box and histogram plots were performed with the R package “ggplot2”. Heatmap was plotted with the R package “gplots”. The survival analysis and Cox proportional hazard regression analysis were carried out on the R package “survival”. The C-index, stepwise Cox analysis, and nomogram were performed with the R package “rms”. LASSO Cox analysis was performed using the R package “glmnet”. The ROC curve was plotted using the R package “qROC”. The DAC analysis was performed using the R package “stdca.R”. The summarized mutation plots were constructed using the R package “GenVisR”. The circus graph was drawn using the “RCircos” package. In the TCGA, LIRI-JP, LICA-FR and GSE1898 datasets, non-HCC patients as well as died within 3 months were removed.

## Supplementary Information

Supplementary Legends.

Supplementary Figures.

Supplementary Tables.

## Data Availability

The data that support the findings of this study are openly available in the TCGA (https://cancergenome.nih.gov/) and ICGC data portal (https://dcc.icgc.org/).
