# Supplementary material for: Identification of multi-omics biomarkers and construction of the novel prognostic model for hepatocellular carcinoma
Source: Sci Rep. 2022 Jul 15;12:12084. doi: 10.1038/s41598-022-16341-w (PMC9287549; doi:10.1038/s41598-022-16341-w)
Supplement: Supplementary file 1 — Supplementary Legends. [file 41598_2022_16341_MOESM1_ESM.docx]

**Supporting legends**

**Figure S1.** **(A, B)** Volcano and heatmap plots show DE-mRNAs between HCC and non-tumour samples. **(C)** 10-fold cross-validation for parameter λ selection. The solid vertical lines are partial likelihood deviance ± standard error (SE). The two dotted vertical lines are drawn at the optimal values by minimum criteria (left) and 1-SE criteria (right). Parameter λ = 0.02808 [log (λ) = -3.573] is chosen via minimum criteria. **(D)** Univariate Cox regression analysis of the six key mRNAs. **(E)** Expression difference of the six key mRNAs between HCCs and non-tumour samples in the TCGA training set. **(F)** Distribution of the mRNA risk score, survival status of HCC patients, and the expression level of the six key mRNAs. HCC, hepatocellular carcinoma; TCGA, The Genome Cancer Atlas; HR, Hazard rate ratio; DE-mRNAs, differentially expressed mRNAs; SE: Standard error; ****, *p*-value < 0.0001.

**Figure S2.** **(A, B)** Volcano and heatmap plots show DE-lncRNAs between HCC and non- tumour samples. **(C)** 10-fold cross-validation for parameter λ selection. Parameter λ = 0.07276 [log (λ) = -2.621 ] is chosen via minimum criteria. **(D)** Univariate Cox regression analysis of the ten key lncRNAs. **(E)** Expression difference of the ten key lncRNAs between HCCs and non- tumour samples in the TCGA training set. **(F)** Distribution of the lncRNA risk score, survival status of HCC patients, and the expression level of the ten key lncRNAs. HCC, hepatocellular carcinoma; TCGA, The Genome Cancer Atlas; HR, Hazard rate ratio; DE-lncRNAs, differently expressed lncRNAs; ****, *p*-value < 0.0001.

**Figure S3. (A, B)** Volcano and heatmap plots show DE-miRNAs between HCC and non- tumour samples. **(C)** Expression difference of the five key miRNAs between HCCs and non- tumour samples in the TCGA training set. **(D)** Distribution of the miRNA risk score, survival status of HCC patients, and the expression level of the five key miRNAs. HCC, hepatocellular carcinoma; TCGA, The Genome Cancer Atlas; DE-miRNAs, differentially expressed miRNA; ****, *p*-value < 0.0001.

**Figure S4. (A)** 10-fold cross-validation for parameter λ selection. Parameter λ = 0.07177142 [log (λ) = -2.634269] is chosen via minimum criteria. **(B)** Copy number alteration of the five key CNV genes in HCCs and non-tumour samples in the TCGA training set. **(C)** Univariate Cox regression analysis of the five key CNV genes. **(D)** Distribution of the CNV risk score, survival status of HCC patients, and the copy number alteration of the five key CNV genes in the TCGA training set. HCC, hepatocellular carcinoma; TCGA, The Genome Cancer Atlas; HR, Hazard rate ratio; CNV, [Copy number variation](https://portal.gdc.cancer.gov/repository).

**Figure S5. (A)** Distributions of various mutation types of the 85 high-frequency SNPs. The histogram at the top indicates the sum of non-synonymous and synonymous mutations in every case. The histogram on the right stands for the sample number suffering from gene mutation. In the heat map, the various colours stand for various mutation types, whereas the white represents no mutation. **(B)** Univariate Cox regression analysis of the ten high-frequency SNPs. **(C)** Distribution of the SNP risk score, survival status of HCC patients, and the mutation status of the seven key SNPs in the TCGA training set. HCC, hepatocellular carcinoma; TCGA, The Genome Cancer Atlas; HR, Hazard rate ratio; SNP, [Single nucleotide polymorphism.](http://www.baidu.com/link?url=jWjHawVwuye4w1uRyloRu5uDK4Bby3Vc-zK3QjDQuCx-Nzm4FA1Nsw21OWc1kunTzC2lhucS7fYnZ7kiV4i3bK&wd=&eqid=bd83a15600044530000000065f4e3b1a)

**Figure S6.** The external validation of the mRNA model. **(A)** Kaplan-Meier survival analysis of the different risk groups stratified with the trisection of the mRNA risk score in the GSE1898 dataset. **(B)** The verification of the mRNA model via the ROC curve and C-index in the GSE1898 dataset.
