# Supplementary figures and images for: Identification of multi-omics biomarkers and construction of the novel prognostic model for hepatocellular carcinoma
Source: Sci Rep. 2022 Jul 15;12:12084. doi: 10.1038/s41598-022-16341-w (PMC9287549; doi:10.1038/s41598-022-16341-w)

Figure S1

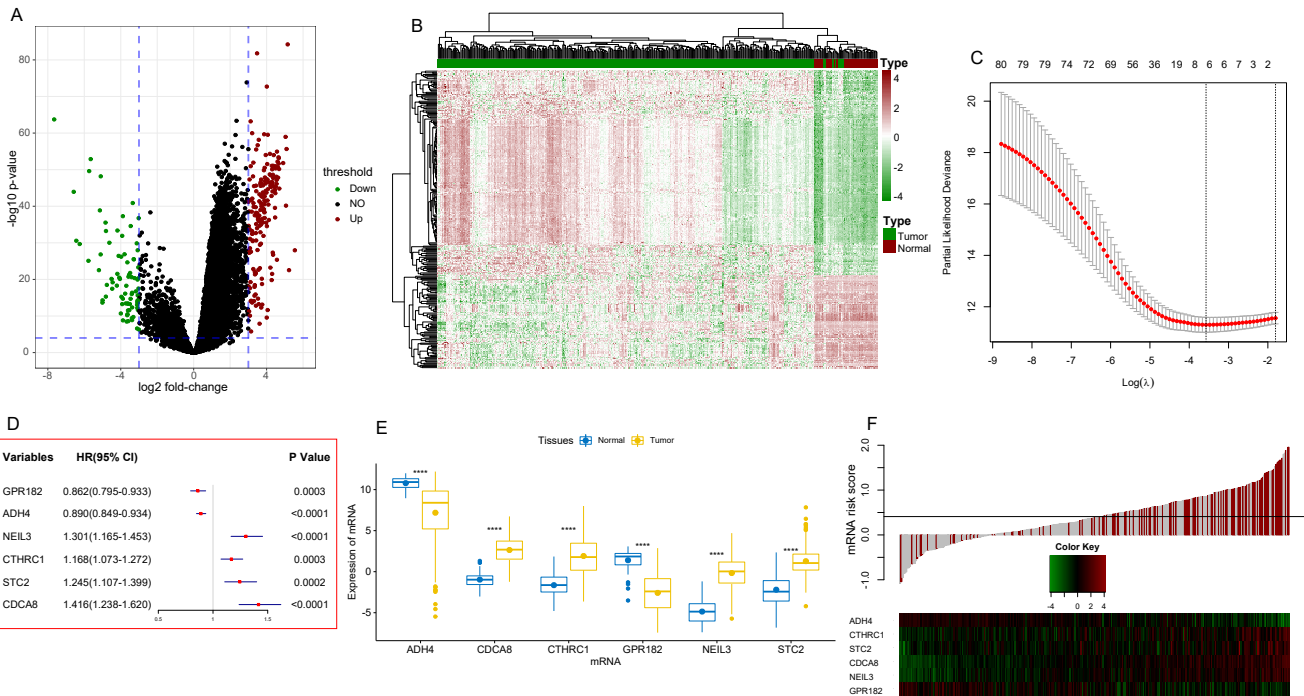

Figure S2

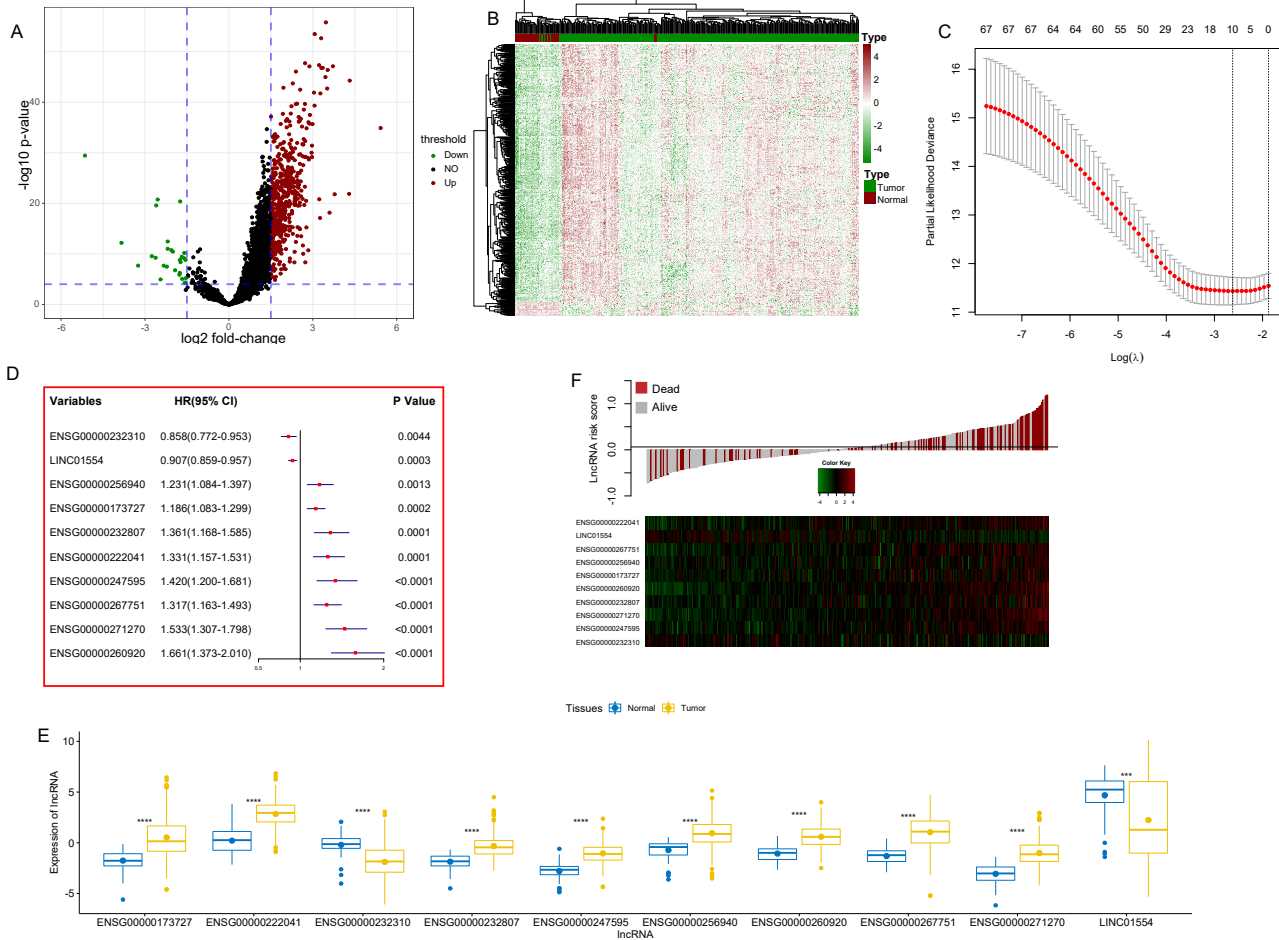



Figure S4

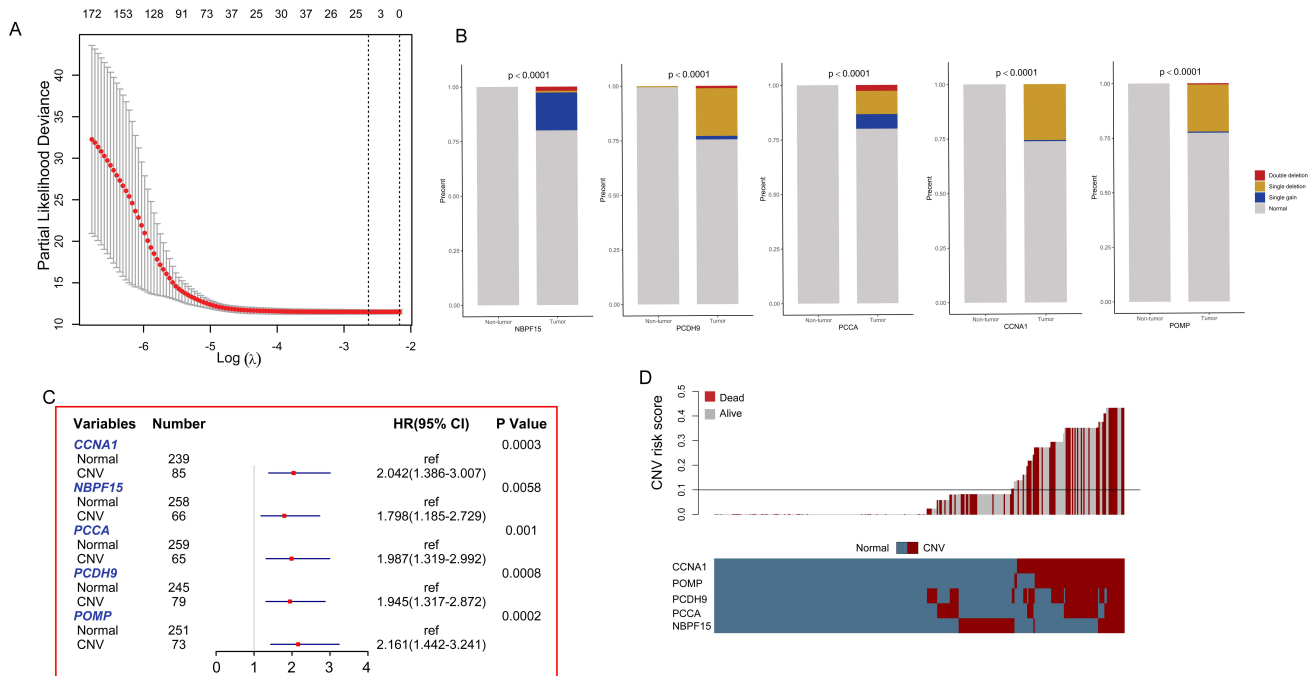

Figure S5

A

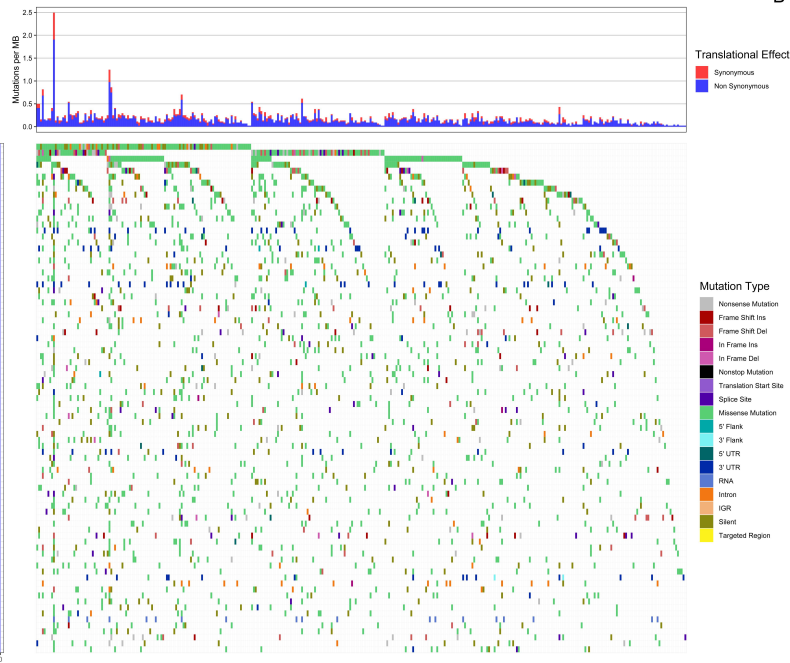

B

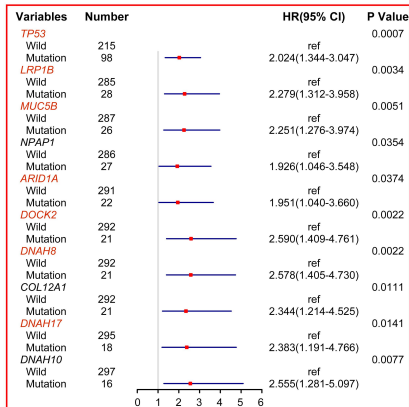

C

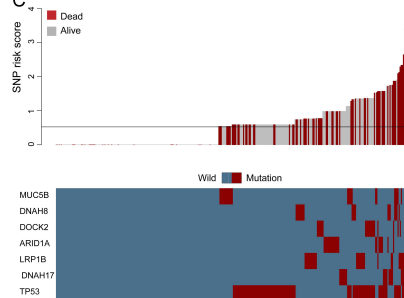

Figure S6

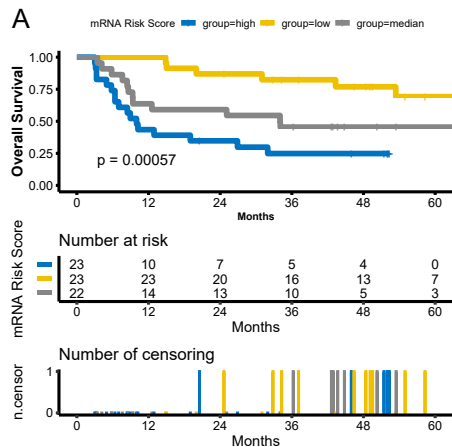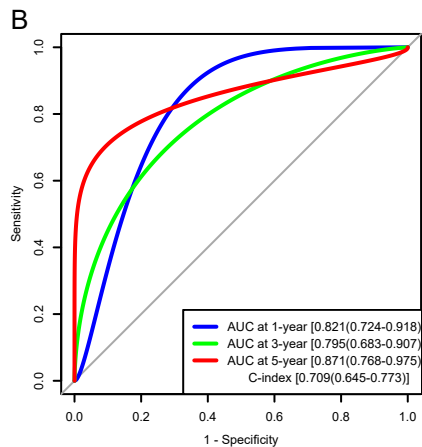

Supplement: Supplementary file 2 — Supplementary Figures. [file 41598_2022_16341_MOESM2_ESM.pdf]
